# Treatment of Gun-shot Injuries of the Head

**Published:** 1865-12

**Authors:** 


### TREATMENT OF GUN-SHOT INJURIES OF THE HEAD.

Dr. John Ashurst, Jr., devotes an article in the *American Journal of Medical Sciences* to the subject of "treatment of gun-shot injuries of the head." From the history of five cases recorded the following conclusions are drawn:

1. In the large number of cases which die under conservative treatment, it does not appear from the autopsies that the use of the trephine could in any way have averted the fatal issue.

2. Many cases which recover without trephining would be seriously jeopardized by rashly admitting the atmosphere to the torn and bruised cranial contents, and thus placing them in the unfavorable circumstances of an open wound, instead of leaving them in a safer position of a sub-cutaneous, or more strictly "sub-osseous" injury.

3. In those cases which recover after the use of the trephine, the instrument does not deserve the credit of the cure; for if there be already an opening through the skull the operation is unnecessary; and if there be not, it adds to the already serious injury a most dangerous complication.

4. There is a close analogy, though often forgotten, between trephining and the resection of long bones. In compound fractures of the extremities we extract loose fragments, restore the others as nearly as possible to their proper places ("setting" the fracture,) and then trust the case to nature. Just so in compound fractures of the skull, it seems to me, we should content ourselves with removing the detached portions of bone, and restoring the rest if possible, by the elevator, or otherwise, to their proper level, and then withhold our hands; conducting the after-treatment upon physiological and rational principles. Trephining is the most serious and fatal of all resections, and I believe the day will yet come when it will be looked upon as a matter of curious and antique surgical history, rather than as an actual and established mode of surgical treatment.

